# Supplementary material for: A metabolic synthetic lethality of phosphoinositide 3-kinase-driven cancer
Source: Nat Commun. 2025 Mar 4;16:2191. doi: 10.1038/s41467-025-57225-7 (PMC11880427; doi:10.1038/s41467-025-57225-7)
Supplement: Supplementary file 1 — Supplementary Information [file 41467_2025_57225_MOESM1_ESM.pdf]

Supplementary Information

Supplementary Table 1
Supplementary Table 2

Supplementary Figure 1
Supplementary Figure 2
Supplementary Figure 3
Supplementary Figure 4
Supplementary Figure 5
Supplementary Figure 6

Supplementary Table 1 - Baseline characteristics and clinical response of patients of the GRAALL03-05/FRALLE2000 cohorts.

	PI3K^{Alt} n = 87 (18%)	PI3K^{Wt} n = 389 (82%)	Overall n = 476	p-value[2]
**Clinical characteristics**				
Age (y)[1]	14.6 (3.2-57.2)	15.3 (1.1-59.1)	15.3 (1.1-59.1)	0.21
Male	66 / 87 (76%)	291 / 389 (75%)	357 / 476 (75%)	0.89
Median WBC (G/l)[1]	**116 (1-770)**	**51 (0-980)**	64 (0-980)	**<0.001**
CNS Involvement	14 / 86 (16%)	37 / 388 (10%)	51 / 474 (11%)	0.082
**Immunophenotype**				**<0.001**
Immature (IM0/δ/γ)	**5 / 75 (7%)**	**84 / 344 (24%)**	89 / 419 (21%)	
IMβ/pre-αβ	33 / 75 (44%)	178 / 344 (52%)	211 / 419 (50%)	
Mature TCRγδ	7 / 75 (9%)	46 / 344 (13%)	53 / 419 (13%)	
Mature TCRαβ	**30 / 75 (40%)**	**36 / 344 (10%)**	66 / 419 (16%)	
ETP phenotype	7 / 59 (12%)	49 / 248 (20%)	56 / 307 (18%)	0.19
**Oncogenetic classification**				**<0.001**
CALM-AF10	4 / 87 (5%)	9 / 389 (2%)	13 / 476 (3%)	
TLX1	5 / 87 (6%)	49 / 389 (13%)	54 / 476 (11%)	
TLX3	7 / 87 (8%)	65 / 389 (17%)	72 / 476 (15%)	
SIL-TAL1	**29 / 87 (33%)**	**28 / 389 (7%)**	57 / 476 (12%)	
Negative	34 / 87 (39%)	185 / 389 (48%)	219 / 476 (46%)	
ND	8 / 87 (9%)	53 / 389 (14%)	61 / 476 (13%)	
**Risk Classifier *N/F/R/P***				
High-risk	72 / 87 (83%)	137 / 389 (35%)	209 / 476 (44%)	**<0.001**
**Treatment Response**				
Corticosensitivity	35 / 83 (42%)	224 / 384 (58%)	259 / 467 (55%)	**0.010**
Chemosensitivity	68 / 85 (80%)	269 / 382 (70%)	337 / 467 (72%)	0.083
Complete Remission	83 / 87 (95%)	357 / 389 (92%)	440 / 476 (92%)	0.37
MRD1 > 10^{-4}	23 / 64 (36%)	100 / 276 (36%)	123 / 340 (36%)	>0.99
Allo-SCT	19 / 84 (23%)	82 / 372 (22%)	101 / 456 (22%)	0.89
**Outcome**				
5-year CIR, SHR [95% CI]	39% [27;49]	27% [22;31]	29% [25;33]	**0.01**
5-year OS, HR [95% CI]	58% [47;70]	74% [70;79]	71% [67;75]	**0.007**

[1]Statistics presented: Median (Minimum-Maximum)
[2]Statistical tests performed: Fisher's exact test; Wilcoxon rank-sum two-tailed test.
p-values < 0.05 are indicated in bold

MRD1 correspond to MRD evaluation after induction and was performed by allele-specific oligonucleotides polymerase chain reaction. T-cell receptor status and oncogenic were performed as described the Methods. T-ALL: T-cell acute lymphoblastic leukemia; WBC, white blood count; CNS, central nervous system; ETP, early thymic precursor; Risk classifier (*NOTCH1/FBXW7-RAS/ PTEN*, *N/F/R/P*) used as described in Trinquand *et al.* ; CR, complete remission; MRD, minimal residual disease; Allo-HSCT, allogenic hematopoietic stem cell transplantation; CIR, cumulative incidence of relapse; OS, overall survival; HR: hazard ratio, SHR: specific hazard ratio, CI: confidence interval

Supplementary Table 2 - Baseline characteristics and clinical response of of R/R T-ALL/LL patients treated with Erwinase/Temsirolimus

Patient	UPNT-1402	UPNT-1401	T-LL-147	T-LL-242	T-LL-244
**Clinical characteristics**					
Age at diagnosis	45 y	50 y	26 y	17 y	8 y
Sex	M	F	F	M	M
**Disease characteristics**					
T-ALL/LL	T-ALL	T-ALL	T-LL	T-LL	T-LL
Oncogenetics	*NA*	NEG	NEG	NEG	NEG
Immunophenotype	*NA*	IMB	pre-AB	*NA*	*NA*
ETP-ALL	0	1	0	0	0
WBC at diagnosis	290	353	-	-	-
CNS involvement	0	0	*NA*	1	0
PI3K alteration	*PTEN$^{Mut+Del}$*	*PTEN^{Del}*	*PTEN$^{Mut+Del}$*	*PIK3R1^{Mut}*	*AKT1^{Mut}*
**Prior treatment history**					
Nb of prior lines of therapy	2[a]	1[b]	3[c]	3[d]	3[e]
Allograft prior ET	yes	yes	no	no	no
**Erwinase-Temsirolimus (ET)**					
Nb of cycles of ET	2	2	2	1	2
Association with Venetoclax	0	1	0	1	1
Response evaluation to ET	MRD^{Neg}	MRD^{Neg}	Volumetric CR	Volumetric CR	Volumetric and Metabolic CR
Toxicity	Grade 3/4	Grade 2/3	Grade 1/2	Grade 1/2	Grade 1/2
Reason for ET discontinuation	Toxicity	Consolidation (DLI)	Consolidation (ASCT)	Supply restriction of Erwinase	Consolidation (ASCT)
Treatments following ET	Temsirolimus	DLI + Venetoclax	ASCT	Temsirolimus + Venetoclax	ASCT
**Evolution**					
Disease evolution after ET discontinuation	T-ALL Relapse	Persistant CR	ARDS post-ASCT	T-LL Progression	Persistant CR
Salvage treatment	Nelarabine	-	-	VPD	
Status	Died from disease progression	Alive (4y after DLI)	Died from ASCT related-toxicity	Died from disease progression	Alive (2 months post ASCT)

Abbreviations : VPD: Vindesine / PEG-asparaginase/Daunorubicin, MRD^{Neg}: MRD negative, ASCT : allogenic stem cell transplantation, CR : complete response, DLI : donor lymphocyte infusion, T-ALL: T-cell acute lymphoblastic leukemia, T-LL: T-cell lymphoblastic lymphoma, IMB: immature beta, pre-AB: pre alpha-beta, ETP: early thymic progenitor, Nb : number, ET : Erwinase / Temsirolimus, *PTEN*^{Mut}: PTEN mutation, *PTEN*^{Del}: PTEN deletion, *PIK3R1*^{Mut}: PIK3R1 mutation, WBC: white blood cells, CNS : central nervous system

a. GRAALL2014[1] (NCT02619630), COOPRALL[2]
b. GRAALL2014[1] (NCT02619630),
c. GRAALL-LYSA LL03[3], Idarubicin/Aracytine, NECTAR[4] (Nelarabine, VP-16, Endoxan)
d. FRALLE T1[5], EuroLB02[6], FRALLE T2[5]
e. CAALL-F01 (NCT02716233), COOPRALL[2], NECTAR[4] (Nelarabine, VP-16, Endoxan)

References

1. Boissel N, Huguet F, Leguay T, et al. In Adults with Ph-Negative Acute Lymphoblastic Leukemia (ALL), Age-Adapted Chemotherapy Intensity and MRD-Driven Transplant Indication Significantly Reduces Treatment-Related Mortality (TRM) and Improves Overall Survival - Results from the Graall-2014 Trial. *Blood*. 2022;140(Supplement 1):112–114.
2. Domenech C, Mercier M, Plouvier E, et al. First isolated extramedullary relapse in children with B-cell precursor acute lymphoblastic leukaemia: Results of the Cooprall-97 study. *Eur. J. Cancer*. 2008;44(16):2461–2469.
3. Lepretre S, Touzart A, Vermeulin T, et al. Pediatric-Like Acute Lymphoblastic Leukemia Therapy in Adults With Lymphoblastic Lymphoma: The GRAALL-LYSA LL03 Study. *J. Clin. Oncol.* 2016;34(6):572–580.
4. Whitlock JA, Malvar J, Dalla-Pozza L, et al. Nelarabine, etoposide, and cyclophosphamide in relapsed pediatric T-acute lymphoblastic leukemia and T-lymphoblastic lymphoma (study T2008-002 NECTAR). *Pediatr. Blood Cancer*. 2022;69(11):e29901.
5. Simonin M, Vasseur L, Lengliné E, et al. NGS-based stratification refines the risk stratification in T-ALL and identifies a Very High-Risk subgroup of patients. *Blood*. 2024;blood.2023023754.
6. Landmann E, Burkhardt B, Zimmermann M, et al. Results and conclusions of the European Intergroup EURO-LB02 trial in children and adolescents with lymphoblastic lymphoma. *Haematologica*. 2017;102(12):2086–2096.

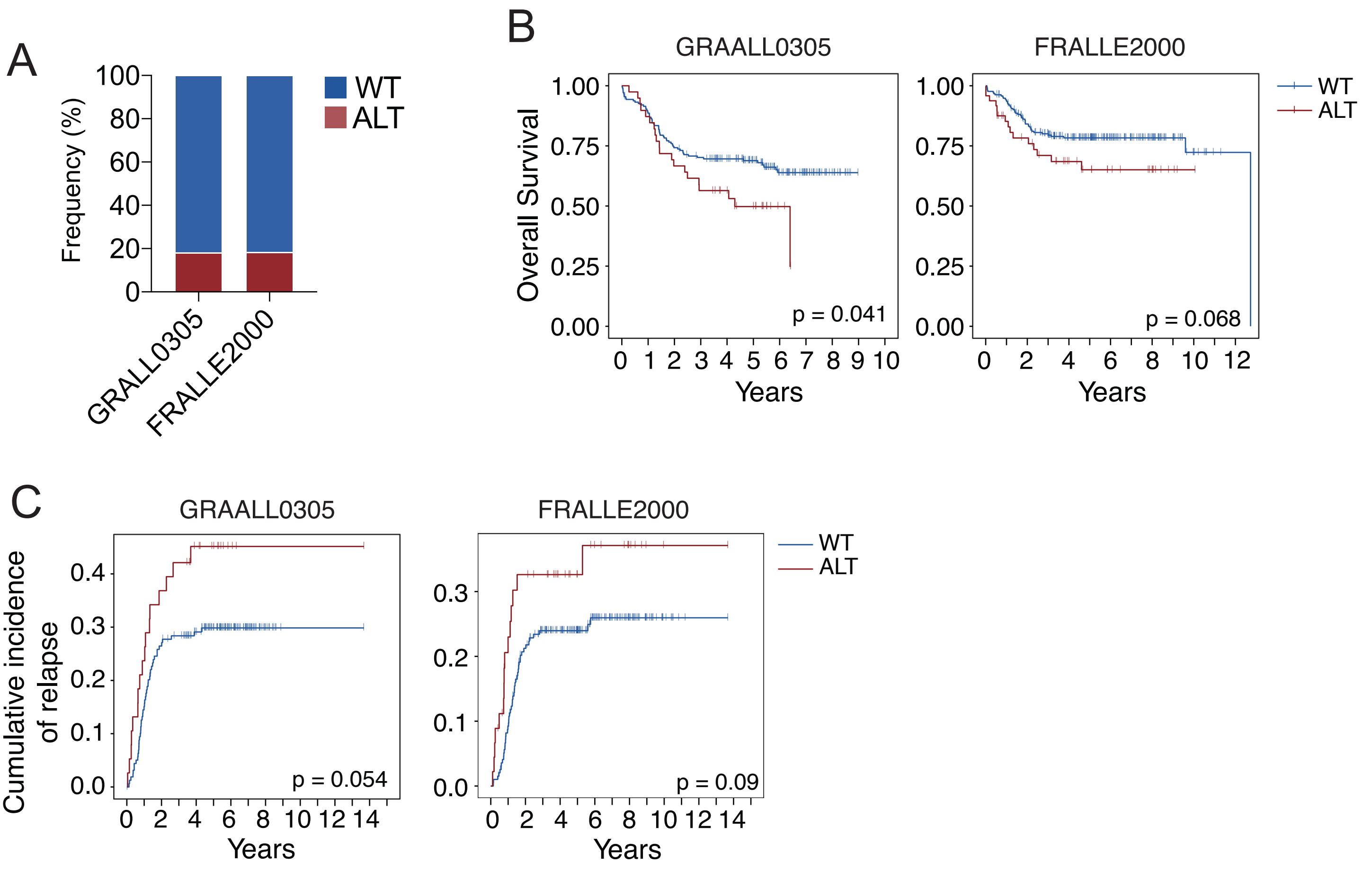

Supplementary Figure 1
A. Incidence of PI3K signaling alterations in the adult (WT: n= 176, ALT: n= 39) and pediatric cohorts (WT: n= 213 , ALT: n= 48).
B. Overall survival of wild-type (WT) and altered (ALT) PI3K signaling patients according to the adult (WT: n= 176, ALT: n= 39) and pediatric cohorts (WT: n= 213 , ALT: n= 48).
C. Cumulative incidence of relapse of wild-type (WT) and altered (ALT) PI3K signaling patients according to the adult (WT: n= 160, ALT: n= 38) and pediatric cohorts (WT: n= 197, ALT: n= 45).

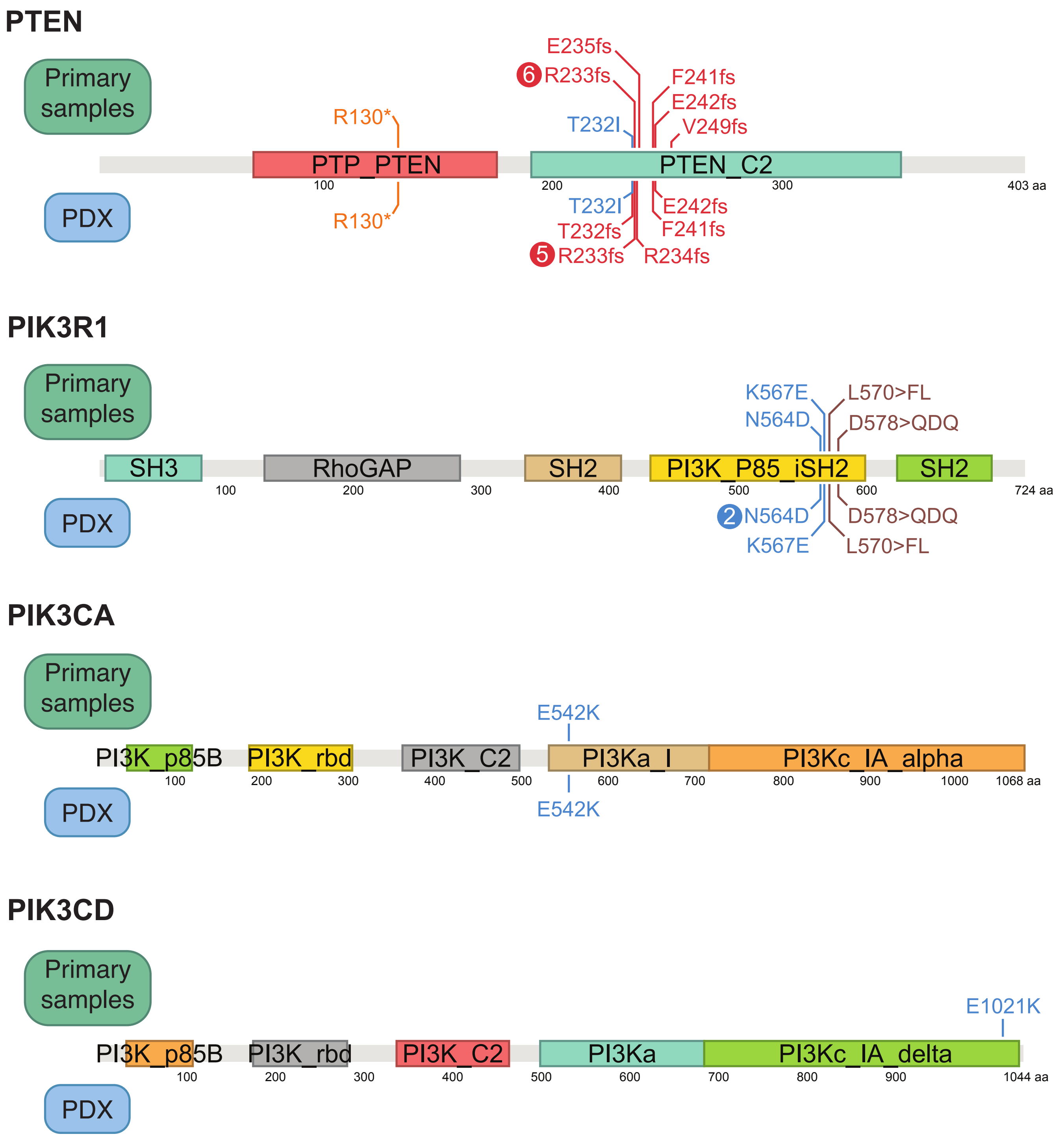

Supplementary Figure 2
Conservation of PI3K signaling core gene mutations evaluated by NGS in primary samples and PDX.

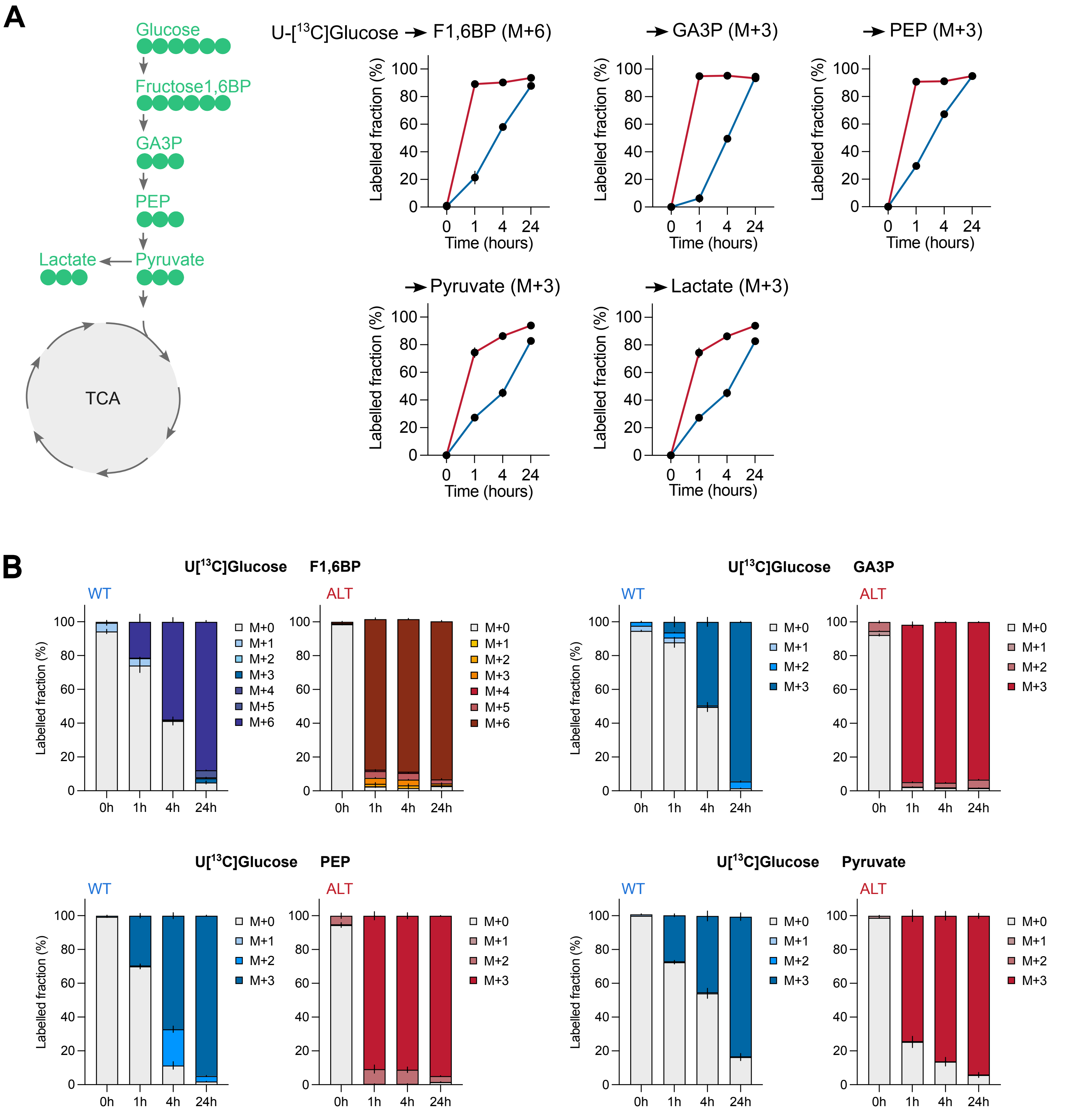

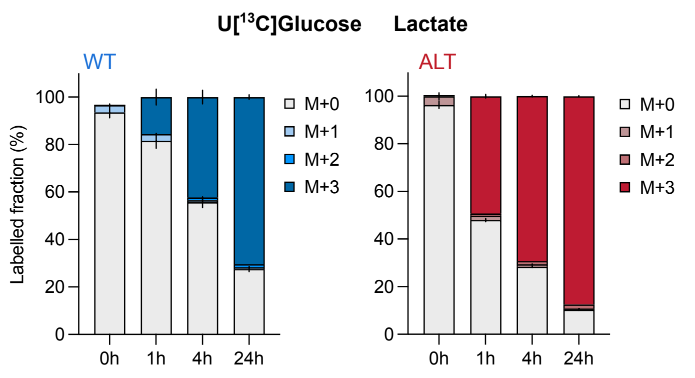

Supplementary Figure 3

A. Left. Schematic representation of metabolic tracing of glycolysis using U-[13C]-glucose. Right. Labeled fraction of glycolysis intermediates from U-[13C]-glucose in wild-type PI3K (n=3 individual PDX) and PI3K-altered (n=3 individual PDX). PDX blasts were cultured in a glucose-free medium for 16h before the addition of 1 mM U-[13C]-glucose for the indicated times. Graphs represent the average value ± SEM of three replicates per sample for the indicated metabolite isotopologues. Blue lines: wild-type PI3K, red lines: PI3K-altered. B. Detailed labeled fractions of the isotopologues of the listed glycolysis metabolites.

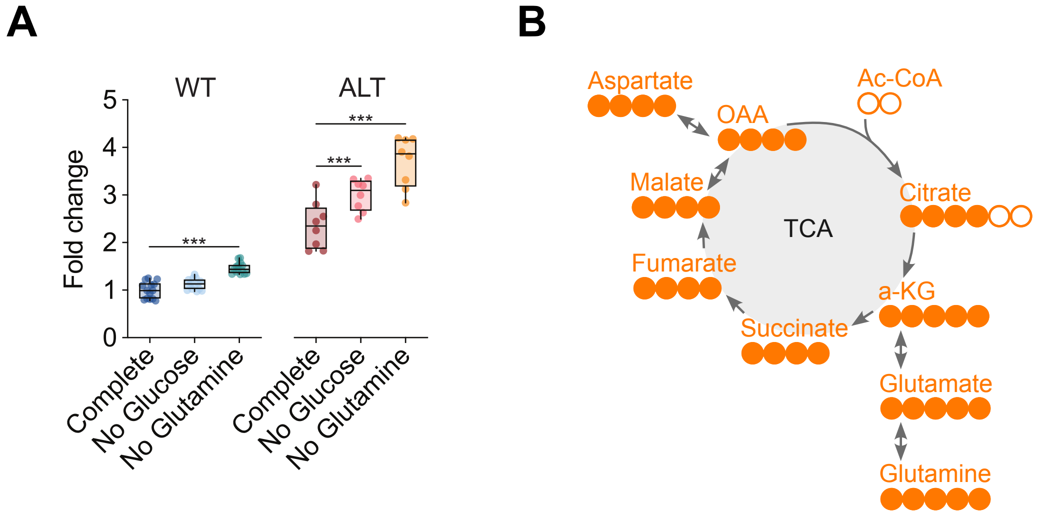

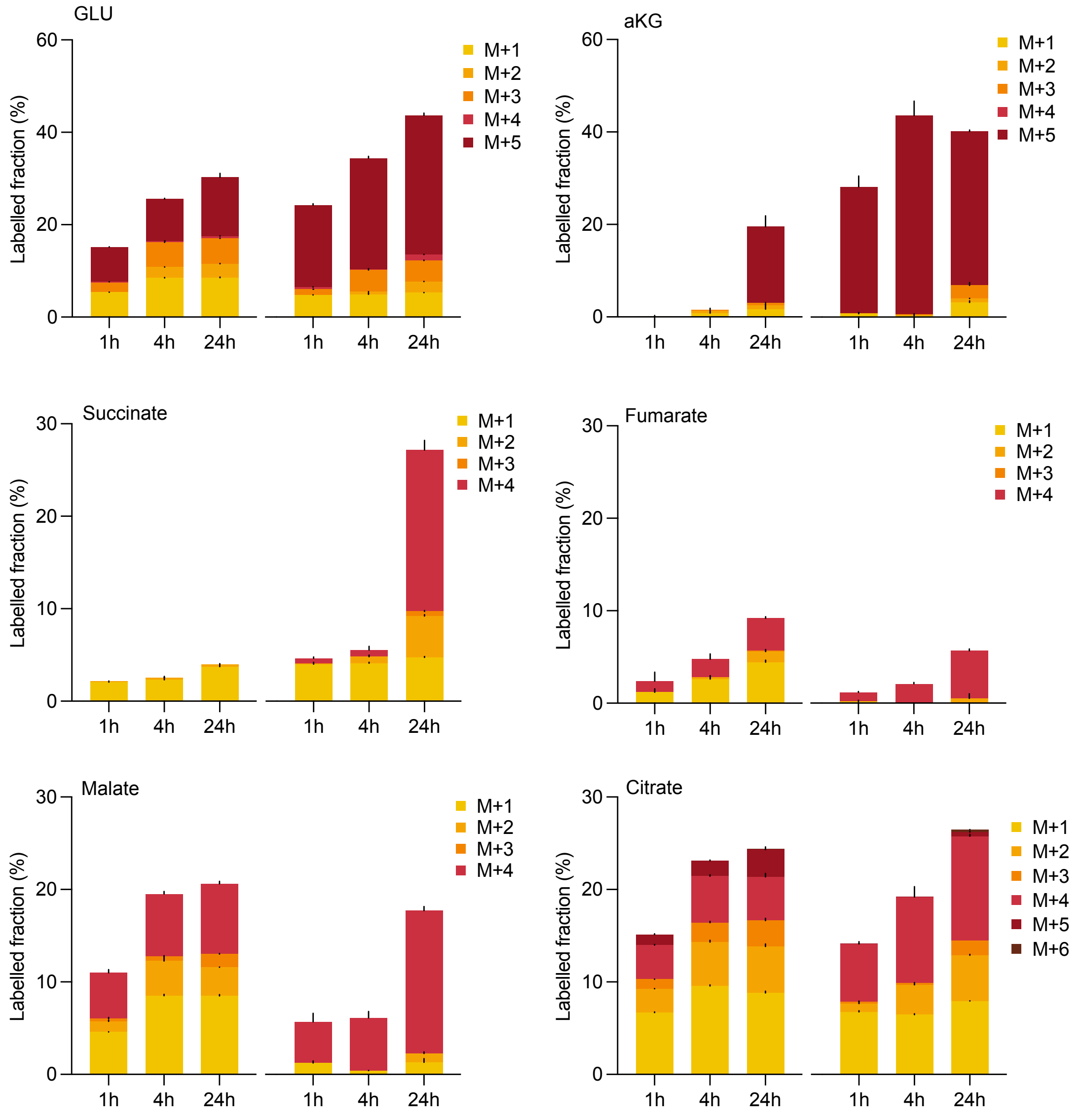

Supplementary Figure 4

A. Glucose consumption in wild-type PI3K (n=14 individual PDX) and PI3K-altered (n=8 individual PDX) blasts was evaluated after 72h of culture in a complete, glucose-free, or glutamine-free medium by metabolomics. Fold changes are indicated. A one-way ANOVA ajusted for multiple comparison (Šídák's multiple comparisons test) was performed. * $p < 0.05$, ***, $p < 0.001$. B. Left. Schematic representation of metabolic tracing of glutaminolysis and TCA using U-[13C]-glutamine. Right. Labeled fraction of glutamate and TCA intermediates from U-[13C]-glutamine in PI3K-altered (n=3 individual PDX). PDX blasts were cultured in a complete or a glucose-free medium for 16h before the addition of 2 mM U-[13C]-glutamine for the indicated times. Histograms represent the average value ± SEM of three replicates per sample for the indicated metabolite isotopologues.

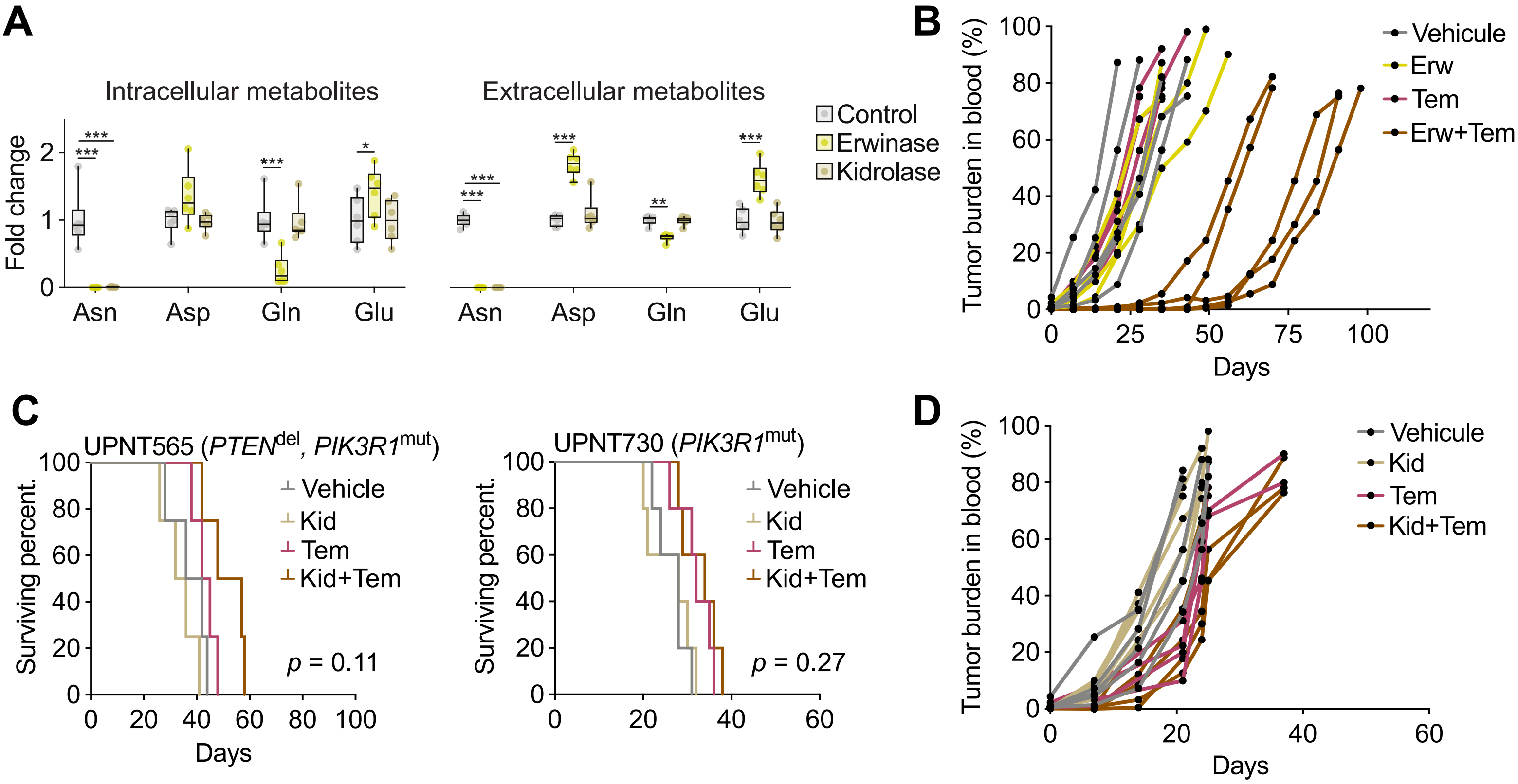

Supplementary Figure 5

A. Fold changes in metabolites detected in PI3K-altered PDX (n=6 individual PDX) and the extracellular medium of cultures or treated with control, erwinase, or kidrolase 1U/ml for 72h. Two-way ANOVA adjusted for multiple comparisons were used (Tukey's tests). *, $p < 0.05$; **, $p < 0.01$; ***, $p < 0.001$). B. Tumor burden evaluated in the circulating blood of mice carrying PI3K-altered T-ALL PDX treated with vehicule, erwinase, temsirolimus, or the combination over time. C. Survival curves of mice xenografted with two PI3K-altered PDX (5 mice/arm/PDX) and treated with vehicule, kidrolase, temsirolimus, or the indicated combinations. D. Tumor burden evaluated in the circulating blood of mice carrying PI3K-altered T-ALL PDX treated with vehicule, kidrolase, temsirolimus, or the combination over time.

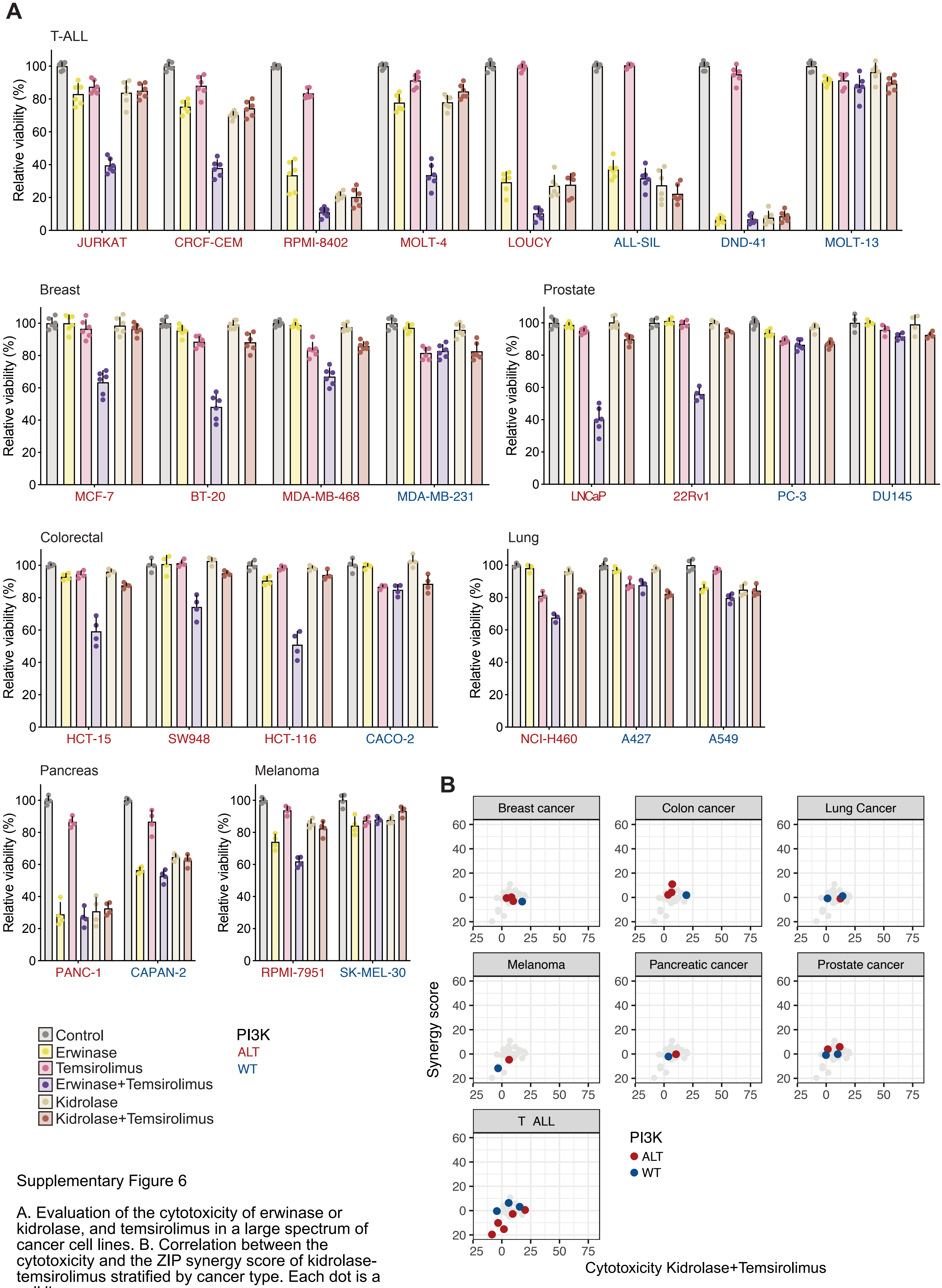

Supplementary Figure 6

A. Evaluation of the cytotoxicity of erwinase or kidrolase, and temsirolimus in a large spectrum of cancer cell lines. B. Correlation between the cytotoxicity and the ZIP synergy score of kidrolase-temsirolimus stratified by cancer type. Each dot is a cell line.
