# Supplementary material for: A metabolic synthetic lethality of phosphoinositide 3-kinase-driven cancer
Source: Nat Commun. 2025 Mar 4;16:2191. doi: 10.1038/s41467-025-57225-7 (PMC11880427; doi:10.1038/s41467-025-57225-7)
Supplement: Supplementary file 2 — Reporting Summary [file 41467_2025_57225_MOESM2_ESM.pdf]

nature portfolio

Corresponding author(s): Vahid Asnafi, Guillaume Andrieu

Last updated by author(s): Oct 28, 2024

Reporting Summary

Nature Portfolio wishes to improve the reproducibility of the work that we publish. This form provides structure for consistency and transparency in reporting. For further information on Nature Portfolio policies, see our Editorial Policies and the Editorial Policy Checklist.

Statistics

For all statistical analyses, confirm that the following items are present in the figure legend, table legend, main text, or Methods section.

n/a	Confirmed	
☐	☒	The exact sample size (*n*) for each experimental group/condition, given as a discrete number and unit of measurement
☐	☒	A statement on whether measurements were taken from distinct samples or whether the same sample was measured repeatedly
☐	☒	The statistical test(s) used AND whether they are one- or two-sided<br>*Only common tests should be described solely by name; describe more complex techniques in the Methods section.*
☐	☒	A description of all covariates tested
☐	☒	A description of any assumptions or corrections, such as tests of normality and adjustment for multiple comparisons
☐	☒	A full description of the statistical parameters including central tendency (e.g. means) or other basic estimates (e.g. regression coefficient) AND variation (e.g. standard deviation) or associated estimates of uncertainty (e.g. confidence intervals)
☐	☒	For null hypothesis testing, the test statistic (e.g. *F*, *t*, *r*) with confidence intervals, effect sizes, degrees of freedom and *P* value noted<br>*Give P values as exact values whenever suitable.*
☒	☐	For Bayesian analysis, information on the choice of priors and Markov chain Monte Carlo settings
☐	☒	For hierarchical and complex designs, identification of the appropriate level for tests and full reporting of outcomes
☐	☒	Estimates of effect sizes (e.g. Cohen's *d*, Pearson's *r*), indicating how they were calculated

Our web collection on statistics for biologists contains articles on many of the points above.

Software and code

Policy information about availability of computer code

Data collection	Excel
Data analysis	R, GraphPad Prism, FlowJo, MetaboAnalyst, GSEA

For manuscripts utilizing custom algorithms or software that are central to the research but not yet described in published literature, software must be made available to editors and reviewers. We strongly encourage code deposition in a community repository (e.g. GitHub). See the Nature Portfolio guidelines for submitting code & software for further information.

Data

Policy information about availability of data

All manuscripts must include a data availability statement. This statement should provide the following information, where applicable:

- Accession codes, unique identifiers, or web links for publicly available datasets
- A description of any restrictions on data availability
- For clinical datasets or third party data, please ensure that the statement adheres to our policy

The high-throughput sequencing data produced were deposited in the European Genome-phenome Archive (EGAD00001010273). Other data generated in this study are not publicly available due to information that could compromise patient privacy or consent but are available upon reasonable request from the corresponding author.

Research involving human participants, their data, or biological material

Policy information about studies with human participants or human data. See also policy information about sex, gender (identity/presentation), and sexual orientation and race, ethnicity and racism.

Reporting on sex and gender	Sex and gender were not considered in the design of the study.
Reporting on race, ethnicity, or other socially relevant groupings	Neither race, ethnicity, nor other socially relevant groupings were considered in the design of the study.
Population characteristics	GRAALL-2003: 15 Years to 59 Years (Child, Adult); GRAALL-2005: 18 Years to 59 Years (Adult); FRALLE2000: Age ≥ 1y and ≤ 10 y. ALLTARGETOBS: 18 Years and older (Adult, Older Adult)
Recruitment	Patients included in this study were enrolled in the GRAALL-2003 (#NCT00222027), GRAALL-2005 (#NCT00327678), and FRALLE-2000 clinical trials. Patients treated with the combination of erwinase and temsirolimus were enrolled in the Registry of Relapsed/Refractory T-cell Acute Lymphoblastic Leukemia (ALLTARGETOBS, # NCT05832125). For the pediatric patient, the off-label use of the treatment has been validated by a specialist committee, and the patient is included in a specific database with authorization to exploit clinical and biological data and written parental consent (IRB #DC-2015-2473, Montpellier University Hospital). Sample collection and analyses were obtained with informed consent under the Declaration of Helsinki with approval from the institutional review boards of institutions that participated in this study.
Ethics oversight	All these details are publicly available here: https://clinicaltrials.gov/study/NCT00222027, https://clinicaltrials.gov/study/NCT00327678, https://clinicaltrials.gov/study/NCT05832125

Note that full information on the approval of the study protocol must also be provided in the manuscript.

Field-specific reporting

Please select the one below that is the best fit for your research. If you are not sure, read the appropriate sections before making your selection.

☒ Life sciences ☐ Behavioural & social sciences ☐ Ecological, evolutionary & environmental sciences

For a reference copy of the document with all sections, see nature.com/documents/nr-reporting-summary-flat.pdf

Life sciences study design

All studies must disclose on these points even when the disclosure is negative.

Sample size	In vivo sample sizes were determined using G*Power. For RNA-seq, we analyzed the entire series available. For metabolomics, two groups of n=7 samples were analyzed after consulting our engineer of the metabolomics core.
Data exclusions	No data were excluded from the study
Replication	Each experiment had a minimum of three replicates. For in vivo data, each arm contained 5 mice to better represent the in vivo variation.
Randomization	Mice were randomly attributed to each treatment arm during in vivo experiments.
Blinding	RNA-seq, metabolomics, and in vivo data were blindly collected and analyzed. For in vitro experiments, blinding was difficult to observe but analyses were carried out with blinding whenever it was feasible.

Reporting for specific materials, systems and methods

We require information from authors about some types of materials, experimental systems and methods used in many studies. Here, indicate whether each material, system or method listed is relevant to your study. If you are not sure if a list item applies to your research, read the appropriate section before selecting a response.

Materials & experimental systems

n/a	Involved in the study
☐	☒ Antibodies
☐	☒ Eukaryotic cell lines
☒	☐ Palaeontology and archaeology
☐	☒ Animals and other organisms
☐	☒ Clinical data
☒	☐ Dual use research of concern
☒	☐ Plants

Methods

n/a	Involved in the study
☒	☐ ChIP-seq
☐	☒ Flow cytometry
☒	☐ MRI-based neuroimaging

Antibodies

Antibodies used	hCD45 V500 (HI30, BD Biosciences), Zombie NIR (BioLegend), pS473-Akt PE (BD Phosflow™ M89-61, BD Biosciences), pS235/236-S6 V450 (BD Phosflow™ N7-548, BD Biosciences), pT36/45-4E-BP1 Alexa Fluor® 647 (BD Phosflow™ M31-16, BD Biosciences)
Validation	hCD45 V500 (HI30, BD Biosciences): The HI30 monoclonal antibody specifically binds to the 180, 190, 205, 220 kDa protein isoforms of CD45.<br>pS473-Akt PE (BD Phosflow™ M89-61, BD Biosciences): The M89-61 antibody recognizes Akt phosphorylated at S473. This phosphorylation site is shared by all three isoforms of Akt.<br>pS235/236-S6 V450 (BD Phosflow™ N7-548, BD Biosciences): The N7-548 monoclonal antibody specifically detects the S6 ribosomal protein phosphorylated at S235 and S236.<br>pT36/45-4E-BP1 Alexa Fluor® 647 (BD Phosflow™ M31-16, BD Biosciences): The M31-16 monoclonal antibody recognizes the phosphorylated T36 and T45 of activated human 4EBP1. The orthologous

Eukaryotic cell lines

Policy information about cell lines and Sex and Gender in Research

Cell line source(s)	ATCC, DSMZ
Authentication	None of the used cell lines were authenticated by our team.
Mycoplasma contamination	Each cell line was monitored weekly for the absence of mycoplasma contamination. In the occurence of such contamination, the cell line was discontinuated and a new mycoplasma-free culture started from frozen stock.
Commonly misidentified lines (See ICLAC register)	

Animals and other research organisms

Policy information about studies involving animals; ARRIVE guidelines recommended for reporting animal research, and Sex and Gender in Research

Laboratory animals	NOD.Cg-PrkdcSCID Il2rgtm1Wjl/SzJ (NSG) mice were purchased from Charles River and maintained in a specific and opportunistic pathogen-free animal facility at the Institut Necker-Enfants Malades. Mice aged from 6 weeks to 12 weeks were used for the in vivo experimentations.
Wild animals	None
Reporting on sex	Both male and female mice were used in our study.
Field-collected samples	None
Ethics oversight	Approved by the French Ministry of Research

Note that full information on the approval of the study protocol must also be provided in the manuscript.

Clinical data

Policy information about clinical studies

All manuscripts should comply with the ICMJEguidelines for publication of clinical research and a completedCONSORT checklist must be included with all submissions.

Clinical trial registration	GRAALL-2003 #NCT00222027<br>GRAALL-2005 #NCT00327678<br>FRALLE-2000

	CAALL-F01 (NCT02716233)<br>Registry of Relapsed/Refractory T-cell Acute Lymphoblastic Leukemia ALLTARGETOBS #NCT05832125
Study protocol	https://clinicaltrials.gov/study/NCT00222027, https://clinicaltrials.gov/study/NCT00327678, https://clinicaltrials.gov/study/NCT05832125
Data collection	GRAALL-2003 #NCT00222027<br>Study Start<br>Primary Completion (Actual)<br>2008-102003-11<br>Study Completion (Actual)<br>2008-12<br>Enrollment<br>300<br><br>GRAALL-2005 #NCT00327678<br>Study Start<br>2006-05<br>Primary Completion (Actual)<br>2014-04<br>Study Completion (Actual)<br>2014-04<br>Enrollment (Actual)<br>1080<br><br>CAALL-F01 #NCT02716233<br>Study Start<br>20016-04<br>Primary Completion (Actual)<br>2026-04<br>Study Completion (Actual)<br>2026-04<br>Enrollment (Actual)<br>1578<br><br>Registry of Relapsed/Refractory T-cell Acute Lymphoblastic Leukemia ALLTARGETOBS #NCT05832125<br>Study Start (Actual)<br>2021-12-14<br>Primary Completion (Estimated)<br>2023-12<br>Study Completion (Estimated)<br>2024-03
Outcomes	All these details are publicly available here:<br>https://clinicaltrials.gov/study/NCT00222027, https://clinicaltrials.gov/study/NCT00327678, https://clinicaltrials.gov/study/NCT05832125<br>https://clinicaltrials.gov/study/NCT02716233

Plants

Seed stocks	This study does not involve plants.
Novel plant genotypes	This study does not involve plants.
Authentication	This study does not involve plants.

Flow Cytometry

Plots

Confirm that:

- ☐ The axis labels state the marker and fluorochrome used (e.g. CD4-FITC).
- ☐ The axis scales are clearly visible. Include numbers along axes only for bottom left plot of group (a 'group' is an analysis of identical markers).
- ☐ All plots are contour plots with outliers or pseudocolor plots.
- ☐ A numerical value for number of cells or percentage (with statistics) is provided.

Methodology

Sample preparation	For proliferation assays, cells were stained with CellTraceTM Violet (BioLegend) before culture. Cells were washed in ice-cold PBS and collected by centrifugation (5 min, 350 x g, 4°C) before staining with Annexin V and propidium iodide in Annexin V binding buffer (BioLegend). For phosflow, cells were washed in ice-cold PBS and collected by centrifugation (5 min, 350 x g, 4° C). Cells were incubated with anti-hCD45 V500 (HI30, BD Biosciences) and Zombie NIR (BioLegend) on ice for 15 min in ice-cold PBS and washed, fixed for 10 min at 4°C and (Fixation Buffer #420801, BioLegend) and washed, then permeabilized 30 min at 4°C (PermBuffer III®, BD Biosciences). After two washes with ice-cold PBS-BSA 0.5%, cells were incubated with anti-pS473-Akt PE (BD Phosflow™ M89-61, BD Biosciences), pS235/236-S6 V450 (BD Phosflow™ N7-548, BD Biosciences) and pT36/45-4E-BP1 Alexa Fluor® 647 (BD Phosflow™ M31-16, BD Biosciences). Fold changes were calculated from relative fluorescence intensities in viable hCD45+ blasts.
Instrument	FACSCanto II
Software	FacsDIVA, FlowJo V10
Cell population abundance	>90%
Gating strategy	hCD45+ Zombie-NIR negative cells were analyzed and the geometric MFI of the indicated markers calculated.

- ☐ Tick this box to confirm that a figure exemplifying the gating strategy is provided in the Supplementary Information.
